# Overexpression of ALDH1 and EMT marker profile are linked with unfavorable outcome in head and neck cancer

**DOI:** 10.4317/medoral.23777

**Published:** 2020-07-23

**Authors:** Rúbia Rocha Vieira, Laura Hildebrand Campos, Luciano Henrique Jesus, Caroline Klabunde, Thiago de Oliveira Gamba, Isadora Luana Flores, Márcia Gaiger Oliveira, Pantelis Varvaki Rados

**Affiliations:** 1 DDS, MSc, Phd. School of Dentistry, Federal University of Rio Grande do Sul, Brazil; 2 DDS. School of Dentistry, Federal University of Rio Grande do Sul, Brazil; 3 DDS, MSc, Phd. School of Dentistry, Caxias do Sul University, Brazil

## Abstract

**Background:**

The aim of this research was to assess the expression of aldehyde dehydrogenase 1 (ALDH1) and epithelial-mesenchymal transition (EMT) markers in head and neck squamous cell carcinoma (HNSCC), and to correlate them with the clinical and histopathological parameters of a patient cohort with follow-up over an 8-year period.

**Material and Methods:**

For this, seventeen HNSCC and non-neoplastic adjacent epithelium (AE) samples were subjected to laser microdissection and real-time PCR to evaluate the mRNA expression of ALDH1, E-cadherin (E-CAD), N-cadherin (N-CAD), and vimentin (VIM). Also, immunohistochemistry was performed for ALDH1, E-CAD, N-CAD, and VIM in the tumor center (TC), invasion front (IF), and AE of the seventeen samples. Mann-Whitney, Kruskal-Wallis and Chi-square tests were used to correlate the mRNA and immunohistochemical expression with different variables, considering *p*<0.05. Kaplan-Meier curves were produced for local recurrence, regional metastasis and treatment.

**Results:**

A mRNA overexpression of ALDH1 in primary tumors was associated with regional metastasis and a high ALDH1 immunostaining was related to metastasis and a worse patient outcome. Additionally, a favorable outcome was associated with the transition phase and an unfavorable outcome was associated with EMT event. An overall 26.9 months was observed with longer survival associated with surgery and radiotherapy.

**Conclusions:**

However, due to the intense variability inherent to the indicator proteins in the EMT process, the complete profile markers related to this biological process should be continuous investigated.

** Key words:**Aldehyde dehydrogenase 1, epithelial mesenchymal transition, head and neck cancer, squamous cell carcinoma, follow-up study.

## Introduction

Head and neck squamous cell carcinoma (HNSCC) is the sixth most common cancer in the world, corresponding to more than 90% of all tumors (1). Despite various studies on carcinogenesis, and attempts to improve the patients’ quality of life, the survival rate has remained low in the last few decades, at around 50% in five years (2,3). This is attributed mainly to regional metastasis, recurrences, and the appearance of new primary tumors (4). Moreover, evidence suggests that a small population of cells, called cancer stem cells (CSCs), play an important role in HNSCC recurrence and metastases (5).

During metastasis, stationary tumor epithelial cells become migratory and invasive. A key event involved in this process is the epithelial-mesenchymal transition (EMT) (6). Once epithelial cells have invaded adjacent tissues, they return to their epithelial phenotype, undergoing an inverse process known as mesenchymal-epithelial transition (MET) (7,8). The EMT and MET processes are dynamic events that can define latent or active intermediate states of tumor, allowing numerous cycles of invasion and metastasis to occur (9).

The aldehyde dehydrogenase 1 (ALDH1) enzyme subfamily consists of three independent members (1A1, 1A2 and 1A3) exhibiting different functions and regulatory effects in different physiological and pathophysiological conditions, including cancer (10). Moreover, ALDH1 has been used as a CSCs marker, and its expression is associated with tumors with greater invasiveness, metastases, treatment resistance, and worse prognosis in HNSCC (10).

The expression of ALDH1 in HNSCC has been previously studied in the tumor center (TC), invasion front (IF), and adjacent non-neoplastic epithelium (AE), showing ALDH1 is useful in identifying tumors with aggressive behavior (11). Moreover, ALDH1 expression was previously correlated EMT phenotypes and considered an epithelial-like marker, nevertheless the exclusively ALDH1 role in the HNSCC cancer correlating with EMT was not previously investigated (12-14). Thus, a better understanding of the other processes involved in carcinogenesis and tumor progression is crucial to understanding the mechanisms used by cells to disseminate and invade (7,15,16).

The aim of this study was to evaluate the mRNA expression of ALDH1, E-cadherin (E-CAD), N-cadherin (N-CAD), and vimentin (VIM) in HNSCC and AE, and their immunoexpression in the TC, IF, and AE. Further, we aimed to correlate the obtained results with clinical parameters, histopathological characteristics, and patients’ outcomes after an 8-year follow-up period.

## Material and Methods

 - Sample description

To perform this study, samples were obtained from specimens during surgical treatment attempts. The samples consisted of 17 paraffin blocks and 34 fresh tissues (17 TC and 17 AE) from the same patients with primary HNSCC in the oral cavity (10 cases), oropharyinx (1 case) and hypopharynx (2 cases vocal cords, 2 cases larynx, 1 case subglottis and 1 case piriform sinus), who did not receive any prior treatment. All patients underwent surgical treatment exclusively.

- Clinical and histopathological characteristics of tumors

Data regarding tumor location and clinical stage were obtained from hospital records. Histopathological grading was performed by two previously trained pathologists, in accordance with Bryne’s criteria (17).

The patients included in this study were divided into two groups in accordance with the follow-up 8 years on: favorable outcome (alive and without recurrence of the tumor), and unfavorable outcome (death by tumor recurrence or metastasis). This time was considered due to the maximum period of follow-up reached to the study.

- Laser microdissection

A cryostat was used to prepare 22 μm thick sections of each sample. The sections were transferred to a pre-sterilized PALM® MembraneSlide (Zeiss®, DEU), briefly heated, and allowed to dry at −20 °C for 2–3 minutes. The slide was then incubated in 70% ethanol for two minutes. Subsequently, staining was carried out in 1% cresyl violet (Sigma-Aldrich®, USA) for 30 seconds, followed by washing with 70% and 100% ethanol. The slides were kept immersed in 100% ethanol until sectioning was carried out, and then dried at room temperature for 1–2 minutes. The microdissection technique was performed with the PALM Microbeam IPZ laser microdissector (Zeiss®, DEU) following the manufacturer's instructions.

- Analysis of mRNA expression

RNA extraction from 34 fresh tissues samples (17 TC and 17 AE) followed the RNA concentration was evaluated by spectrophotometric reading at 260 and 280nm wavelengths. The estimation of the degree of purity was through the A260/A280 ratio and 4 µL with 2µg (0.5 µg/µL) of total mRNA per sample were used to synthesis of cDNA from previously microdissected areas using the RNeasy Micro kit (Qiagen®, USA) and the SuperScript® VILO™ cDNA Synthesis Kit (Invitrogen®, USA), respectively, following the manufacturers’ instructions. The cDNA samples obtained were then subjected to real-time PCR to analyze the mRNA expression of markers of the EMT process and ALDH1.

TaqMan® primers for E-CAD (Hs01023894_m1), N-CAD (Hs00983056_m1), VIM (Hs00185584_m1), and ALDH1 (Hs00946916_m1) from Life Technologies®, USA, were used for the mRNA expression analysis. All reactions were performed in triplicate and normalized to GAPDH (Hs99999905_m1) and β-actin (Hs99999903_m1) (Life Technologies®, USA). A previously tested sample was used as a positive control, while ultrapure water was used as the negative control including real-time PCR reagents with no mRNA material. All reactions were performed in a Step One Plus® thermocycler (Life Technologies®, USA).

- Immunohistochemical quantification and reproducibility

The immunohistochemical staining for each marker was evaluated in the different areas of interest by two previously trained examiners using light microscopy with a final amplification of 400X. Cells with brown staining, irrespective of the intensity of the marking, were considered positive for E-CAD (membrane; Cell Signaling; Clone 24E10; 1:200), N-CAD (cytoplasm and nucleus; Abcam; Clone AB18203; 1:300), and VIM (membrane; Dako; Clone V9; 1:100). The score was 1 (0–50 % of immunopositive cells) or 2 (51–100% of immunopositive cells) (11). As for ALDH1 (cytoplasm; DB Biosciences; Clone 44; 1:50), the score was 1 (<5% of immunopositive cells) or 2 (≥5% of immunopositive cells) (11).

Reproducibility was confirmed during the course of the study. For every 10 slides evaluated, one was randomly selected for re-evaluation after a period of 7 days (Kappa > 0.7).

- Analysis of the EMT process in tumor samples and classification of AE

The tumor samples were classified as low E-CAD expression and high N-CAD and/or VIM expression, high E-CAD expression and low N-CAD and/or VIM expression, or transition phase (tumors that did not fit the score criteria). For the AE, a qualitative definition was used: normal (high E-CAD expression, and low N-CAD and VIM expression) or altered (when the immunostaining revealed distinct patterns) (6,18). The immunohistochemical scores were considered to classification tumors low E-CAD expression and high N-CAD (E-CAD – Score 1/N-CAD-Score 2), high E-CAD expression and low N-CAD (E-CAD – Score 2/N-CAD-Score 1), transition (E-CAD – Score 1/N-CAD-Score 1 or ECAD-Score 2 NCAD-Score 2).

- Statistical analysis

A Chi-square test was used to verify the association between the immunohistochemical expression and the other variables. The mRNA expression analysis and its relationship to the study variables were compared using the Mann-Whitney or Kruskal-Wallis test using the SPSS software, version 21 (IBM Corporation, USA) (significance level set at *p*<0.05). Life Tables were used to calculate survival rates at 6, 18, 24, 36, and 48 months after diagnosis with a complete follow-up of 8 years. Kaplan-Meier curves were produced for local recurrence, regional metastasis and treatment with a confidence interval of 95%.

## Results

The clinical and histopathological parameters of the samples are summarized in Table 1. The mean age in the favorable outcome group was 53.3 years (range 39–70), and that in the unfavorable outcome group was 60.2 years (range 48–72). The majority of individuals in both groups were male (85.7% and 100%) and Caucasian (57.1% and 90%). Regarding alcohol consumption and tobacco use, the majority in the favorable outcome group were alcohol drinkers and smokers (57.1%), while in the unfavorable outcome group most were former drinkers (60%) and former smokers (80%). Furthermore, the majority of individuals in the favorable outcome group had tumors of sizes T1 and T2 (85.7%), with an absence of regional metastasis (71.4%) and located in the oral cavity (57.1%). In the unfavorable outcome group, there was a prevalence of tumors of a larger size (T3 and T4), with the presence of regional metastases, and located in the oral cavity, all with a rate of 60% (Table 1).

**Table 1:**
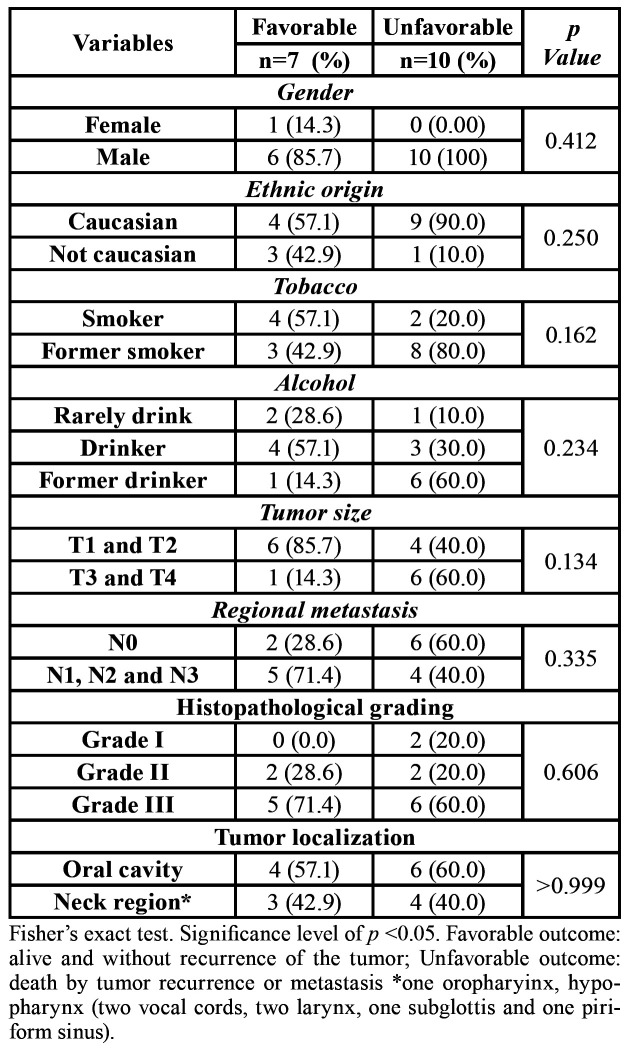
Sample description according to patients’ outcome with follow-up of 8 years.

- Survival by local recurrence 

**Figure 1:**
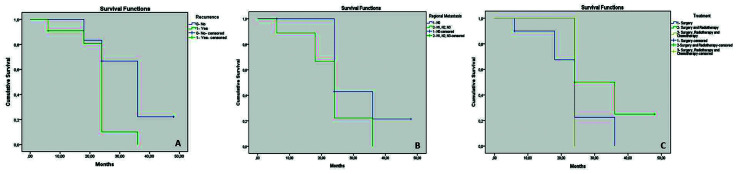
Survival curves. A. Survival by local recurrence. A longer survival rates of HNSCC patients with no recurrence was reached (No:36.6 months/Yes:22.9 months). B. Survival by regional metastasis. Note a longer survival rates of HNSCC patients with absence of regional metastasis was observed (N0: 31.7 months/N1, N2 and N3: 23.3 months). C. Survival by treatment. Note, the HNSCC patients treated with surgery and radiotherapy presented longer survival rates (33 months) comparative with surgery, radiotherapy and chemotherapy (24 months) and exclusively surgery (23.5 months).

The mean overall duration of survival was 26.9 months (range: 6 to 48 months), and the survival rates at 6, 18, 24, 36 and 48 were: 0.9, 0.8, 0.6, 0.2 and 0, respectively. The mean survival time was longer for no recurrence (36.6 months, 95% CI: 25.2 to 42.1) followed by with recurrence (22.9 months, 95% CI: 18.6 to 27.3) (Fig. 1), but this was not significant (chi square (1) = 3.726, *p*=0.054).

- Survival by regional metastasis

The mean overall duration of survival was 26.9 months (range: 6 to 48 months), and the survival rates at 6, 18, 24, 36 and 48 were: 0.8, 0.6, 0.4, 0.2 and 0, respectively. The mean survival time was longer for N0 (31.7 months, 95% CI: 24.2 to 39.2) followed by N1, N2 and N3 (23.3 months, 95% CI: 17.3 to 29.3) (Fig. 1), but this was not significant (chi square [1] = 2.733, *p*=0.09).

- Survival by treatment

The mean overall duration of survival was 26.9 months (range: 6 to 48 months), and the survival rates at 6, 18, 24, 36 and 48 were: 0.9, 0.67, 0.36, 0.25 and 0, respectively. The mean survival time was longer for surgery and radiotherapy (33 months, 95% CI: 24.7 to 41.3) followed by surgery, radiotherapy and chemotherapy (24 months, 95% CI: 24 to 24) and surgery (23.5 months, 95% CI: 17.8 to 29.33) (Fig. 1), but this was not significant (chi square [2] = 3.148, *p*= 0.207).

- mRNA expression 

ALDH1 mRNA overexpression in the tumor samples was associated with the presence of regional metastasis (*p*=0.021). E-CAD overexpression in the tumor was associated with a favorable evolution (*p*=0.051) (Table 2).

**Figure 2:**
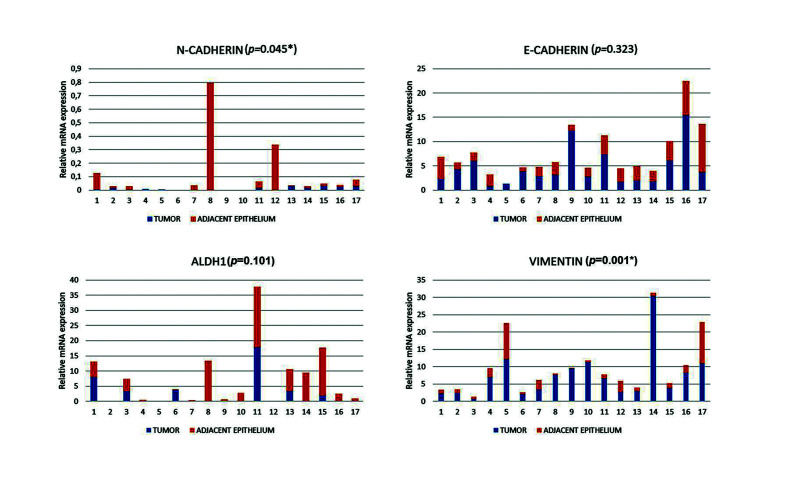
Relative mRNA expression in the tumor and adjacent epithelium (AE) of head and neck squamous cell carcinoma (HNSCC) samples. N-CAD was overexpressed in the AE (*p*=0.045). E-CAD expression was not significant *p*=0.323. ALDH1 expression was not significant (*p*=0.101). VIM was overexpressed in the tumors (*p*=0.001).

N-CAD was overexpressed in the AE when compared to the tumor (*p*=0.045). E-CAD and ALDH1 expression were not significant *p*=0.323 and (*p*=0.101). Conversely, VIM was overexpressed in the tumors when compared to the AE (*p*=0.001) (Fig. 2).

**Table 2:**
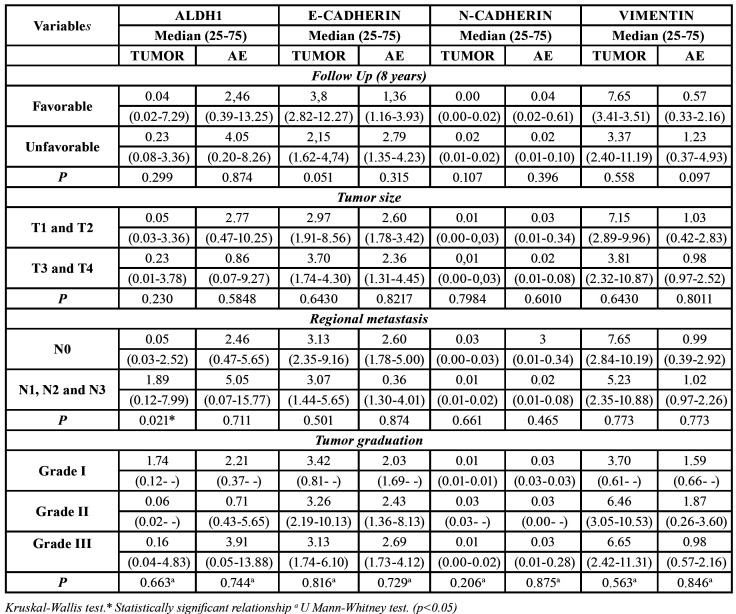
Relationship among patients follow-up, tumor size, regional metastasis, tumor gradation and mRNA.

- Immunohistochemical findings

During the immunohistochemical processing, three samples were excluded, leaving the final sample count of 14 individuals. ALDH1 overexpression was associated with an unfavorable outcome in both the IF (*p*=0.023) and TC (*p*=0.031). ALDH1 overexpression in the IF was associated with the presence of regional metastasis (*p*=0.031) (Table 3).

**Figure 3:**
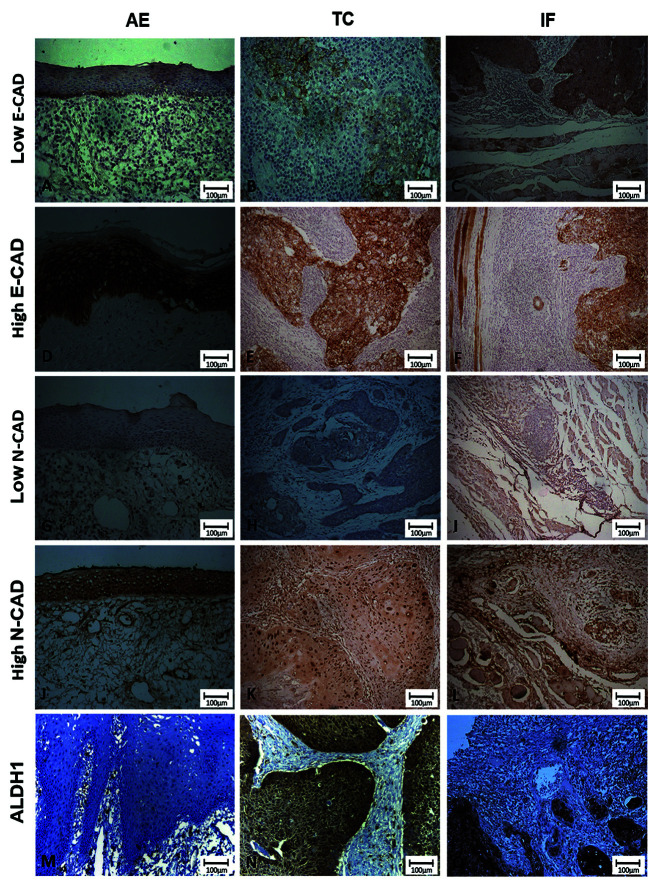
Immunostaining profile of E-CAD, N-CAD, and ALDH1 in the adjacent epithelium (AE), tumor center (TC), and invasive front (IF) of head and neck squamous cell carcinoma (HNSCC) samples. Note the tumor patterns classified as epithelial-mesenchymal transition (EMT) (low E-cadherin, high N-cadherin) (B, C, K, and L) and mesenchymal-epithelial transition (MET) (high E-cadherin, low N-cadherin) (E, F, H, and I). ALDH1 presents evident positivity in the TC and IF of HNSCC samples.

E-CAD overexpression in the TC was associated with an unfavorable patient outcome (*p*=0.031) (Table 3). Low E-CAD immunoexpression was associated with altered epithelia (*p*=0.027) (data not shown). Fig. 3 illustrates the complete immunoexpression findings.

Regarding VIM, a score of 1 was observed in 100% of the three areas studied. Because it is a constant variable, no statistical analysis was performed (data not shown).

- Low E-CAD and High N-CAD/High E-CAD and low N-CAD or transition phases in IF and TC

In the IF, one sample was classified as low E-CAD and high N-CAD, ten as transition phase, and three as high E-CAD and low N-CAD. A significant association between a low expression of E-CAD and the low E-CAD and high N-CAD/transition phases, as well as between a high expression of this protein and high E-CAD and low N-CAD (*p*=0.011), was seen. Additionally, an association was also observed between the classified histopathological grading and low E-CAD and high N-CAD, as well as between the moderate and transition phases, and between the poor outcome and high E-CAD and low N-CAD (*p*=0.003) (Table 4).

In the TC, one sample was classified as low E-CAD and high N-CAD, eight as transition phase, and five as high E-CAD and low N-CAD. An association was found between a favorable outcome and the transition phase, as well as between an unfavorable outcome and low E-CAD and high N-CAD/ high E-CAD and low N-CAD (*p*=0.048). Another significant association was found in relation to E-CAD expression, in which low expression was associated with low E-CAD and high N-CAD /transition phase, and overexpression with high E-CAD and low N-CAD (*p*=0.003) (Table 4).

**Table 3:**
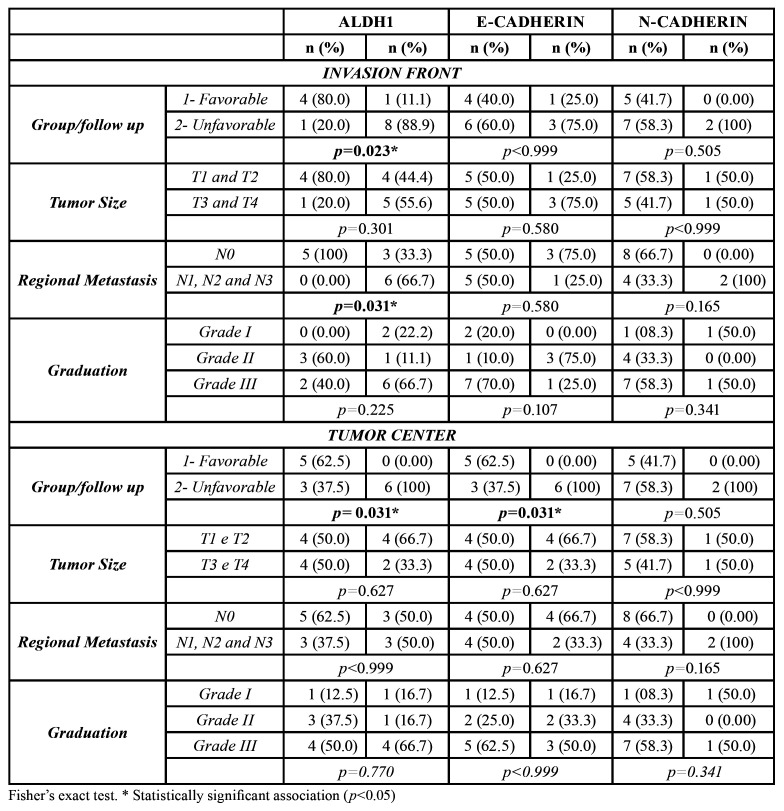
Association among ALDH1, E-CAD, N-CAD immunoexpression in tumor areas and clinical parameters.

**Table 4:**
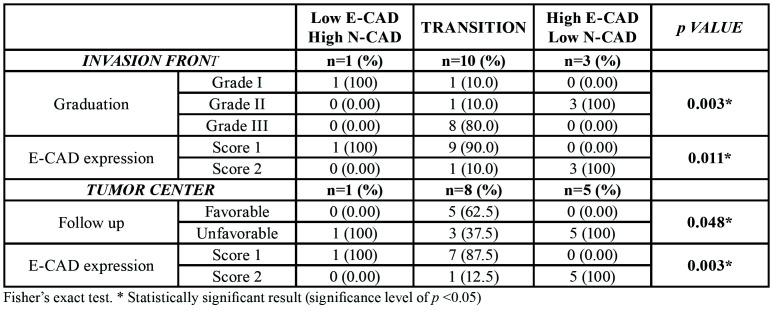
Association between EMT process (low E-CAD and high N-CAD/transition/high E-CAD and low N-CAD) in tumor areas and studied variables.

## Discussion

To our knowledge, this is the first study on solid tumors to have evaluated the expression of ALDH1 and molecules involved in the EMT process in different areas of HNSCC and AE, by isolating the epithelial tissue from the connective tissue. In addition, a descriptive analysis of the results of mRNA expression with the immunostaining of the studied proteins was conducted in the present study. However, there was no access to matching samples of lymph node metastasis from their cohort in order to evaluate the expression of ALDH1 and EMT markers. Also, a convenience sample including different anatomic sites was used which presents as a weakness of study.

The demographic profile of the participating individuals, and the clinical characteristics of the tumors, were similar to those of different consulted studies (19,20). In addition, a large part of the patients had been exposed to habits considered risk factors for the development of HNSCC (21). A complete recorded prospectively data as from 2009 until 2017 was conducted, and all patients’ data were round renewed until the last follow-up date accessed by researchers. Our overall survival was similar to the literature, and this may be reflection of clinical status and treatment. Also, a survival analysis by recurrence, regional metastasis and treatment was included. The overall 26.9 months survival was observed considering local recurrence, regional metastasis and therapy treatment established. Surgery and radiotherapy presented better results (33 months) followed by surgery, radiotherapy and chemotherapy (24 months) and surgery (23.5 months). Moreover, patients with local recurrence (No:36.6/Yes:22.9) and regional metastasis (N0: 31.7/N1, N2 and N3: 23.3) presented shorter survival. Our survival parameters are comparable than previous literature and reinforce the role of adjuvant therapies such as radiotherapy with an increase of survival rates when comparable exclusively surgery (3,4).

In our study, ALDH1 mRNA overexpression and immunoexpression were associated to the presence of regional metastases. The presence of ALDH1 in cancer cells with a greater invasive capacity has already been described (11,22,23). Likewise, previous studies have reported the association between EMT and CSCs markers and ALDH1 expression in primary tumors and regional metastasis (12,13,24-26). This suggests that ALDH1 positivity can be associated to cells in the EMT process and, consequently, to metastasis (24,26). Furthermore, high ALDH1 immunoexpression, regardless of the assessed tumor area, was associated with an unfavorable outcome, suggesting this marker could be used as an indicator of worst prognosis for patients with HNSCC (24,27). Moreover, high ALDH1 expression was recently associated with a high intensity of tumor budding. This reinforces the role of CSCs in carcinogenesis through ALDH1 expression, which can accelerate the invasion of cancer cells and, consequently, worsen the biological tumor behavior (28).

Regarding E-CAD in the AE, we observed low mRNA expression and the preservation of immunostaining, suggesting that the non-neoplastic epithelial tissue expresses adequate levels of E-CAD protein in regions of cell adhesion, without necessarily entailing oscillation in mRNA expression. The opposite situation was found in the tumor, where there was a high level of mRNA expression and low protein immunostaining, suggesting that a higher production of mRNA does not necessarily translate into protein synthesis by the neoplastic cells. Similar results were observed in the N-CAD gene and in immunostaining in the AE and VIM in the tumor, wherein an intense production of mRNA does not necessarily determine high protein synthesis. However, the low expression of E-CAD and the overexpression of N-CAD in the AE can suggest that the initial gene deregulation observed through mRNA expression with no evident modification in protein synthesis is crucial to the loss of adhesion of the epithelium. Thus, these findings suggest the suspicious of field cancerization with no morphological modification.

Analyzing the results of the relationship between the adhesion molecule immunoexpression and the clinical parameters, we observed an association between high E-CAD immunoexpression in the TC and an unfavorable outcome in patients, a result similar to that found in breast tumors (29). However, N-CAD and VIM immunoexpression did not show the same relationship regardless of the evaluated region, which suggests these markers are not relevant to prognostic evaluation in the studied sample.

The EMT process in tumors is a dynamic and complex biological process, leading to the need to evaluate its intermediary stages individually (19). In the present study, we evaluated the association between cases classified as low E-CAD and high N-CAD, transition, high E-CAD and low N-CAD, and different variables. A significant association was observed between low E-CAD immunoexpression in tumors and EMT/transition phases, corroborating published findings according to which cells do not need to gain mesenchymal features in the initial EMT process (18). Therefore, more important than the gain of mesenchymal properties is the loss of E-CAD expression that directs the malignant cell to initiate the EMT process.

In the IF, the transition phase was related to undifferentiated tumors, supporting the results of previous studies that associated an intermediate EMT state with undifferentiated cells or cells exhibiting CSCs properties (18,25). In addition, the existence of CSCs at the IF was previously reported (30). Moreover, it may be difficult to interpret the results of the transition phase due to its plasticity, whereby a migratory cell can express both epithelial and mesenchymal properties.

Evaluating the EMT process in the TC, we observed an association between the low E-CAD and high N-CAD/ high E-CAD and low N-CAD phases and an unfavorable patient outcome, while the transition phase was related to a favorable outcome. This distinct behavior can be explained by the fact that each cancer type has a distinct propensity to exhibit diverse EMT states (18). Nieto *et al*. [2016] reported that these different EMT states may explain the inconclusive clinical significance of EMT. The authors suggest the EMT process is a focal event, occurring in relation to the local microenvironment of the tumor cells (18,26). Besides, there is a cellular hierarchy within a tumor population, with neoplastic cells in distinct stages of proliferation and differentiation, which can affect the assessment of the EMT process through immunostaining (18). Thus, the influence of the EMT process on cancer progression and patient survival is not completely understood.

According to the obtained results, it can be concluded that ALDH1 overexpression is associated to regional metastasis and a worse outcome in HNSCC patients based on clinical and microscopical parameters. However, the same conclusion should be carefully drawn when considering EMT mechanisms, due to the high variability inherent in the process, suggesting the need for further studies to investigate the prognostic value of these mechanisms.
